# Research progress in the estimation of the postmortem interval by Chinese forensic scholars

**DOI:** 10.1080/20961790.2016.1229377

**Published:** 2016-12-13

**Authors:** Chengzhi Li, Qi Wang, Yinming Zhang, Hancheng Lin, Ji Zhang, Ping Huang, Zhenyuan Wang

**Affiliations:** a School of Forensic Science and Medicine, Xi'an Jiaotong University, Xi'an, China; b Shanghai Key Laboratory of Forensic Science, Shanghai Forensic Service Platform, Institute of Forensic Science, Ministry of Justice, PRC, Shanghai, China

**Keywords:** Forensic science, forensic pathology, postmortem interval, methods, Chinese

## Abstract

The determination of time since death or the postmortem interval (PMI) is one of the most important and frequently asked questions in forensic medicine. Medicolegal scholars and forensic pathologists around the world have studied the estimation of PMI extensively in the past, and many novel methods and advanced technologies have now been applied in the field. For several centuries, Chinese forensic examiners have also worked on the estimation of the PMI, and there are a large number of excellent studies published in Chinese rather than in English, and these are not easily accessible or known internationally. Therefore we have conducted a review of relevant studies published by Chinese forensic scholars in the last few decades. The scope of this review is to provide a concise summary of the current progress in the estimation of PMI by Chinese forensic researchers using molecular biology, spectroscopic technology, entomological methods, energy changes, thanatochemistry and other methods.

## Introduction

The determination of the time since death or postmortem interval (PMI) is one of the most important and most frequently asked questions in forensic practice [1]. It is also one of the fundamental tasks of the forensic pathologist who is consulted when a body is found. From the point of view of criminal law, a precise estimation of the PMI enables verification of witness statements, thus limiting the number of suspects and possible alibis. Imprecise or incorrect estimation of PMI may confuse and complicate an investigation [2]. From 1984 to 2015, Chinese forensic scholars have made great progress in improving the estimation of PMI. Many methods have been applied to help determine the time since death, and these can be divided into the following categories: molecular biology methods (degradation of DNA, RNA or proteins); spectroscopic technology (Fourier transform infrared or Raman microspectroscopy); entomological methods (either a carrion insect development or a succession model [3]); estimation of energy changes in the body after death (cooling or blood ATP levels); thanatochemistry methods (describing changes in the chemical composition of various body fluids [4]); and other methods such as imaging technology, electrophysiological methods and enzyme activity. Despite the fact that studies on the estimation of PMI span decades, there is still a long way to go before many of these can be applied definitively in forensic practice. The scope of this review is to provide a concise summary of the progress made by Chinese forensic scholars in improving PMI estimation methods.

## Molecular biology methods

### Studies on the estimation of PMI based on RNA degradation

After an organism's death, RNA is degraded by ribonucleases present in the cell, as well as those originating from bacteria or other environmental sources [5]. In general, mRNA is thought to be more unstable than DNA and other proteins. However, after crucial methodological advances in RNA extraction, reverse transcription, and the invention of real-time quantitative PCR, a number of forensic laboratories have monitored RNA degradation to estimate PMI [6]. In 2013, Young et al. studied the time-dependent differences in RNA decay rates [7]. They found that a segment of β-actin RNA from tooth pulp can be used to estimate PMI for pigs buried within a shallow grave for up to 84 days. In recent years, Chinese forensic scholars have also investigated PMI estimation based on RNA degradation, and their work is summarized in Table 1.

**Table 1.** Studies by Chinese forensic scholars on PMI estimation based on RNA degradation.

Year	Authors [reference number]	Sample source	Tissues and organs	Temperature (°C)	RNA	PMI
2005	Xiao Junhui et al. [8]	Rat	Myocardium and diaphragm muscle	21	β-actin mRNA	12 d
2007	Liu Ji et al. [9]	Rat	Brain	15, 20	GAPDH mRNA,18S rRNA	7 d
2007	Chen Xiaorui et al. [10]	Rat	Retina	20	β-actin,Pgk1,and Rpl4 mRNA	28 h
2009	Ren Guangmu et al. [11]	Rat	Brain and spleen	20	GAPDH mRNA, β-actin mRNA	12 d
2010	Wu Hongyan et al. [12]	Rat	Liver	10, 25	GAPDH mRNA	48 h
2011	Liu Yuelin et al. [13]	Rat	Brain,heart and kidney	20	β-actin mRNA	96 h
2013	Wang Hui et al. [14]	Rat	Cardiac muscle, liver, brain and skeletal muscle	—	miR-122, miR-133a, miR-150, miR-195, miR-206	48 h
2014	Li Wencan et al. [15]	Rat	Heart	—	18S rRNA and microRNA	7 d
2014	Lv Yehui et al. [6]	Rat	Spleen	4 or 25	mRNA, microRNA, 18S rRNA and U6 snRNA	260 h

In the past, few studies were conducted using RNA degradation to estimate PMI because it was difficult to extract the RNA. However, as we show in Table 1, Chinese researchers working on this subject for the last 10 years have found that measuring RNA degradation after death is especially useful for precise PMI estimation, but as all the experimental samples have come from rats, we do not know if this data can be extrapolated to humans. Another limitation is that RNA degradation studies are often carried out at a fixed temperature, so this does not reflect the effects of changing environmental conditions on RNA degradation. Furthermore, all of these studies were conducted over relatively short time-frames, using soft tissue samples that gave highly variable results [7]. The scope for the application of RNA degradation in forensic practice is still unresolved.

### Studies on the estimation of PMI based on DNA degradation

When an organism dies, internal cellular nucleases cause chromosomal DNA to degrade into smaller fragments over time. As the PMI lengthens, chromatin is degraded until no high molecular weight DNA (HMW-DNA) remains [16]. DNA degradation as a predictor of the PMI has been studied for more than 40 years [17]. In Table 2, we summarize the studies by Chinese scientists on the use of DNA degradation to estimate PMI.

**Table 2.** Studies by Chinese forensic scholars on PMI estimation based on DNA degradation.

Year	Authors [reference number]	Sample source	Tissues and organs	Temperature (°C)	Detection methods	PMI
2000	Liu Liang et al. [18]	Rat	Brain	22–28	Image analysis	24 h
2001	Liu Liang et al. [19]	Rat	Kidney	16–22	Auto-TV-image system	48 h
2002	Chen Yuchuan et al. [20]	Human	Marrow in bosom bone	20–25	Feulgen staining and computerized image analysis	7 d
2005	Zhang Lan et al. [21]	Rat	Liver, kidney and spleen	20	Terminal deoxynucleotide transferase	48 h
2005	He Fanggang et al. [22]	Human	Spleen	4, 17–28	Feulgen staining and image analysis technology	7–36 h
2008	Hu Jun et al. [23]	Rat	Bone marrow and brain	10, 20	Single cell gel electrophoresis	40 h
2011	Li Shanshan et al. [24]	Human	Liver	10, 20, 30	Image analysis technique	13–34 h

DNA degradation rates are influenced by many factors such as temperature, pH values and diseases among others. To overcome the effect of temperature, Larkin et al. investigated the effect of accumulated degree-days on the DNA yield from skeletal muscle and its possible application to estimating the PMI [16]. The rate of DNA degradation varies in different tissue (liver, kidney and spleen) and in samples from different organisms (rat or human). Unfortunately, in such studies the extraction and quantitative analysis of DNA are usually performed under strictly controlled conditions and a small error can lead to inaccurate results. This work is also time-consuming and expensive. Additionally, most of the data obtained can only be used effectively for short PMI estimations. Therefore, DNA degradation is considered to be of limited value to forensic investigations requiring an estimation of PMI.

### Studies on the estimation of PMI based on protein degradation

Protein is a basic cellular component of organisms, found in all tissues and organs. When life ends, cellular proteins degrade under the influence of various proteolytic enzymes. Technological advances have allowed researchers to apply a range of methods to study the relationship between protein degradation and PMI [25–29]. We have summarized the Chinese research on PMI estimation using protein degradation in Table 3.

**Table 3.** Studies by Chinese forensic scholars on PMI estimation based on protein degradation.

Year	Authors [reference number]	Sample source	Tissues and organs	Protein	Detection methods	PMI
1994	Huang Qiuju et al. [30]	Human	Blood	Complement 3 (C3)	Cross immunoelectrophoresis	40 h
2004	Lv Jiangming et al. [31]	Rat	Cardiac and skeletal muscle	Actin	Immunohistochemical S-P method	54 h
2006	Zheng Xudong et al. [32]	Human	Skeletal muscle	Troponin I	Western blot technique	5 d
2006	Wu Rongqi et al. [33]	Rabbit	Skeletal muscle	Myofibril fragmentation index	Biuret method	48 h
2007	Bian Jie et al. [34]	Human	Cardiac and skeletal muscle	Myoglobin	Western blot and imaging technique	72 h
2008	Kuai Jinxia et al. [35]	Rat	Cardiac muscle and lung	Tubulin	Western blot	7 d
2008	Liu Yang et al. [36]	Rat	Cardiac muscle, liver, spleen, lung, kidney, brain and skeletal muscle	Actin	Western blot	168 h

Huang et al. studied the complement 3 (C3) cleavage of blood from human cadavers [30]. They found that the higher the temperature and the longer the time-frame, the faster the C3 cleavage; there was also a significant positive correlation between the C3 cleavage and the PMI. Lv et al. found that the extent of HHF35-staining depletion in cardiac and skeletal muscle cells increases with increased PMI within a certain range [31]. Zheng et al. described that troponin I content in the human pectoralis muscle gradually decreased with the extension of the PMI [32]. During the same year, Wu et al. reported that the myofibril fragmentation index significantly increased with prolonged PMI [33]. Bian et al. reported the rate of degradation of human myoglobin in both skeletal and cardiac muscles [34]. Kuai et al. found that the levels of tubulin in cardiac muscle and lung tissue of rats varied with PMI [35]. However, Liu et al. found that there was a strong correlation between actin degradation and PMI, and the coefficient of determination (*R* ^2^) exceeded 0.75 in the cardiac muscle, liver, spleen, lung, kidney, brain and skeletal muscle of rats [36].

The rate of protein degradation is similar for DNA and RNA, and the protein degradation curve often follows a parabola or a straight line. Because of their intrinsically stable structure, some proteins are commonly used as markers for PMI estimation, including actin, tubulin and thyroglobulin [35–37]. Though specific proteins have a significant correlation with PMI, the process of protein degradation is still affected by environment temperature and putrefying bacteria, which complicates the application of this method in forensic practice.

## Spectroscopic techniques

Fourier transform infrared (FTIR) spectroscopy is one of the most powerful methods for recording IR spectra of biological materials. It is rapid and yields a strong signal with only a few micrograms of sample, because the penetration depth of IR is independent from sample thickness [38]. Because of recent technological developments in instrumentation, Raman microscopy has emerged as a powerful analytical tool in biology [39]. The resolution of confocal Raman microscopy (CRM) is on the submicron scale, close to 200 nm using a laser in the visible wavelength region [40]. In addition, it is not sensitive to water content in samples [39]. Many Chinese forensic scholars have also applied spectroscopy to PMI estimation (Table 4).

**Table 4.** Application of spectroscopy technology in PMI estimation by Chinese forensic scholars.

Year	Authors [reference number]	Sample source	Tissues and organs	Spectroscopic technology	PMI
2009	Huang Ping et al. [41]	Rat	Liver and spleen	FTIR	144 h
2010	Huang Ping et al. [42]	Rat	Cardiac muscle	FTIR	168 h
2010	Huang Ping et al. [43]	Rat	Spleen	FTIR	72 h
2010	Xiong Ping et al. [44]	Human	Kidney and liver	Raman microspectroscopy	48–72 h
2011	Huang Ping et al. [45]	Human and rat	Liver, spleen, kidney, heart, etc.	FTIR	168 h
2011	Xiong Ping et al. [46]	Human	Liver	Laser confocal micro-Raman	48–72 h
2011	Guo Ping et al. [47]	Human	Spleen	Raman microspectroscopy	48–72 h
2012	Li Shiying et al. [48]	Rat	Spleen	FTIR	15 d
2012	Ke Yong et al. [38]	Rat	Brain	FTIR	144 h

Huang et al. reported that the quantitative analysis of FTIR spectra related to PMI shows a strong linear correlation between absorbance ratios and increasing time after death [41]. Following this, Huang et al. estimated PMI in cardiac muscle and spleen tissue using FTIR spectroscopy [42,43]. Xiong et al. found that the relative peak intensities (*I*_1094_/*I*_2923_) of confocal Raman microscopy for the tissue cells decreased gradually with increased PMI from 48 to 72 h after death [44]. They then analyzed rat and human tissues using FTIR spectroscopy [45]. They found no obvious changes in the position of the absorbance bands for either rat or human, which both provided similar results. Xiong et al. found that there was no significant change for the position of the main scattering peaks of the liver tissue within PMI between 48 and 72 h, but the intensity of these peaks were significantly different [46]. The nucleic acid-related peak (1094 cm^−1^) significantly decreased in intensity with increased PMI. The intensity of the lipid-related peaks (1454 cm^−1^, 2923 cm^−1^) showed no significant changes, but each relative peak (*I*_1094_/*I*_2923_) reduced intensity over time. Also using Raman spectroscopy, Guo et al. corroborated this, reporting that the relative peak intensities (*I*_1094_/*I*_2923_) for spleen tissue gradually decreased with increased PMI over a range of 48–72 h [47]. In particular, peaks related to nucleic acids (1094 cm^−1^) were observably reduced.

Li et al. examined rat's liver and spleen tissue using FTIR [48]. Ke et al. also described the changes in attenuated total reflection-Fourier transform infrared (ATR-FTIR) spectra when analyzing rat brain from 0 to 144 h post mortem [38]. They found a significant linear correlation between the relative absorption intensity and PMI.

Spectroscopy presents several advantages as a new method for the estimation of PMI. It is more convenient and easier to carry out than other methods, and just a few micrograms of sample are enough for detection. Various types of spectroscopic techniques have been applied, such as fluorescence spectroscopy [49], spectrophotometric analysis [50], laser-induced breakdown spectroscopy [51] and UV-induced autofluorescence [52], but as we show in Table 4, much of this type of work has also been attempted by Chinese researchers to estimate early PMI. However, the results are highly influenced by environment factors such as temperature, humidity and wind. Therefore, further study is needed before spectroscopic technology could be used routinely in forensic practice.

## Entomological methods

Forensic entomologists have primarily used either a carrion insect development or an insect succession model to infer time since death [3]. The more common of the two is to use insect development, in which a model is used to determine the age of the carrion insects found on or near the corpse [53–57]. This value then provides a minimum PMI under the assumption that oviposition or larviposition occurred on the deceased immediately following death [3]. Chinese studies in this field are summarized in Table 5.

**Table 5.** Studies on estimation of PMI by Chinese forensic scholars with entomology.

Year	Authors [reference number]	Experimental premise
1994	Tang Zhizhou et al. [58]	Length of maggots
2000	Niu Qingshan et al. [59]	Relationship between the sum of effective temperature and the length of larvae or weight pupa of *Lucilia sericata*
2002	Wang Jiangfeng et al. [60]	Chronometrical morphology of *Aldrichina grahami*
2005	Liu Xingjia et al. [61]	Growth time of the maggot fly
2007	Zhu GH et al. [62]	Puparial case hydrocarbons of *Chrysomya megacephala*
2010	Shi Yanwei et al. [63]	Development of *Chrysomya megacephala* (Diptera: Calliphoridae)
2010	Chen Lushi et al. [64]	Necrophagous flies life cycle
2011	Chen Lushi et al. [65]	Sarcosaphagous flies community composition, seasonal variation and growth of the length
2013	Ma Ting et al. [66]	Growth and development patterns of *Hydrotaea spinigera* (Diptera: Muscidae)
2013	Liu Ying et al. [67]	Morphologic observation of *Boettcherisca peregrina*
2014	Xu Hong et al. [68]	Cuticular hydrocarbons of larvae in *Aldrichina grahami*
2014	Feng Dianxing et al. [69]	Pupal stage of *Megaselia scalaris*
2015	Yang Yongqiang et al. [70]	Development of *Hemipyrellia ligurriens* (Wiedemann) (Diptera: Calliphoridae) at constant temperatures

Tang et al. formulated an equation for estimating the PMI by measuring the length of maggots [58], then in 2000 Niu et al. established three linear regression equations between the sum of effective temperature and the length of larvae or weight of pupa of *Lucilia sericata* (*K*_1_ = 2.088 0 + 0.801 4*X*_1_; *K*_2_ = 54.091 7 − 2.881 4*X*_2_; *K*_3_ = 133.218 0 − 2.631 2*X*_3_) [59]. Wang et al. found that structural traits of posterior spiracles, skin and digestive dust consistently changed with larval growth and could be used as larval age markers [60]. Liu et al. reported a direct connection between the length of maggot and the host's time of death [61]. They deduced a formula, with relevance ratio θ, for calculating the PMI using the length of the maggots. Zhu et al. reported that cuticular hydrocarbon was a potential indicator of the weathering time in *Chrysomya megacephala*, and possibly for other necrophagous flies, and might also be used to determine the PMI [62]. Shi et al. found that the maximum length of larvae and the weight of pupae increase stepwise with increasing malathion concentrations through the period of larval development [63]. Also in 2010, Chen et al. found that the abundance of necrophagous flies at high temperatures in summer was greater than at low temperatures in winter [64]. Later on, Chen et al. obtained base data of the sarcosaphagous fly community composition, and seasonal changes in maggot growth rates in the suburbs of Guiyang [65]. Ma et al. found that the relationship between larval body length and time, at four different constant temperatures could be simulated by a logistic function y=(a+bx)/[1+exp(c+dx)][66]. Xu et al. developed a mathematical model derived from multivariate linear regression analysis, for determining age of the larvae based on age-dependent changes in cuticular hydrocarbons [68]. The same year, Feng et al. found that some morphological features that changed during development within the puparium could be used as age markers [69]. Yang et al. constructed isomegalen and isomorphen diagrams depicting the time of larval length and developmental event, respectively, at different temperatures [70]. A thermal summation model was also constructed via regression analysis, by estimating the developmental threshold temperature (*t*) and the thermal summation constant (*K*).

Entomological methods are mainly used for the estimation of longer PMI. Though entomology plays an important role in the estimation of PMI, it still has some disadvantages. The results are often confounded by investigator subjectivity, such as seasonal and regional factors. International contribution of forensic entomologists has meant that many insects have been identified as being useful for determining PMI [54,55,71,72]. Many new techniques have also been employed for this research, including GC-MS [73], optical coherence tomography [56], artificial neural networks [74] and virtual forensic entomology [75] to explore the relationship between carrion insects and PMI.

## Estimation of PMI based on energy changes

The changes in energy of a decaying organism can be monitored using corpse temperature or blood ATP levels. In general, the most accurate time-of-death estimates are obtained from the measurement of postmortem cooling [76]. Because of its central role in energy metabolism, adenosine-5′-triphosphate (ATP) is a conserved and highly specific marker that can be useful for determining the PMI with varying causes of death [77]. In Table 6 we list the Chinese studies which report using energy change measurements to estimate PMI.

**Table 6.** Reports by Chinese forensic scholars in which PMI estimation depends on the changes in energy.

Year	Authors [reference number]	Basic novelty
1984	The Chinese PMI estimation working group [78]	Relationship between rectal temperatures of 581 corpses and PMI
2004	Wang Qiong et al. [79]	Relationship between temperatures of liver, rectal and ear and PMI
2011	Sun Tingyi et al. [80]	Relationship between blood ATP level and PMI
2012	Yang Yulei et al. [81]	Correlation between factors related to body temperature and PMI
2013	Mao Shiwei et al. [77]	Estimation of PMI depending on the changes in ATP and its degradation products
2013	Yang Tiantong et al. [82]	Correlation between ATP concentration in human venous blood and PMI under various ambient temperatures
2014	Sun Tingyi et al. [83]	A mathematical model using rabbits to characterize the correlation between blood ATP levels in the right ventricle and PMI

Chinese forensic scholars measured the rectal temperatures of 581 corpses aged 1–80 years [78]. They constructed numerous regression formulae for PMI estimation, and found that the environmental temperature, warm clothing and how the corpse was placed have important effects on the body temperature drop. A decade later, Wang et al. explored the relationship between PMI and the temperatures of the liver, rectum and ear [79]. They found that the measurement of rectal temperature is more precise than the others within 4 h, and the measurement of liver temperature is most reliable between 4 and 24 h. Sun et al. found that rabbit blood ATP levels at 25 °C rises in the early period after death, and it reaches its peak at 8 h after death [80]. It then decreases as PMI extends. There is clearly an effective correlation between blood ATP level and PMI in the range of 8–56 h after death. Yang et al. constructed a statistically significant regression model for the relationship between factors related to decreasing body temperature and PMI [81]. The equation they formulated is

$$y=25.993+0.04x_{1}+0.172x_{2}+0.88x_{3}+0.047x_{4}+0.373x_{5}+0.347x_{6}-0.766x_{7}$$

where $x_{1}$ = fat thickness; $x_{2}$ = environment temperature; $x_{3}$ = warm clothing; $x_{4}$ = object for parking corpse; $x_{5}$ = ventilation conditions; $x_{6}$ = cause of death; = rectal temperature.

Mao et al. found that the *K* value is a robust index for the estimation of PMI ($K_{v}=100\times (H_{x}+H_{x}R)/(\mathrm{ATP}+\mathrm{ADP}+\mathrm{AMP}+\mathrm{IMP}+H_{x}R+H_{x})$), based on highly significant linear correlations between PMI and concentrations of ATP breakdown products [77]. The same year, Yang et al. found that blood ATP level decreased with PMI extension [82]. They obtained six two-variable cubic curve equations (with *R* ^2^ from 0.976 to 0.990) after regression analysis, and formed a surface equation after interpolation analysis. And they also obtained the three-variable quadratic surface equation. Sun et al. developed a mathematical model using interpolation functions to characterize the correlation between the blood ATP level in the right ventricle of rabbit and PMI at different ambient temperatures [83].

Chinese forensic scholars have also constructed numerous regression formulae for the estimation of PMI based on the body cooling process. Yet environmental factors, especially ambient temperature, are still central problems to be solved for this method to be used in future forensic practice. Considering these problems, researchers in other countries have taken advantage of climatic-control chambers to explore the environmental effects on the estimation of PMI [76]. In addition, they have also made use of accumulated degree-days to account for the effect of frequent temperature changes [84]. Blood ATP levels are also affected by physical health, cause of death and environmental temperature. Thus, discovering a more precise method for using energy changes in the estimation of the PMI is still the goal for all forensic scholars.

## Thanatochemistry methods

In the past, a variety of chemical methods have been employed to determine time since death [85–90]. Thanatochemistry refers to changes that occur in the chemical composition of various body fluids immediately after death [17]. A number of forensic scholars have tried to define the relationship between PMI and postmortem biochemical changes in various body fluids such as blood, serum, cerebrospinal fluid, vitreous humour and synovial fluid [4]. In Table 7 we show the Chinese forensic studies on the estimation of PMI with thanatochemistry methods.

**Table 7.** Studies by Chinese forensic scholars on PMI estimation based on thanatochemistry methods.

Year	Authors [reference number]	Sample source	Tissues and organs	Test material	Temperature (°C)	PMI
2002	Zhang Yanling et al. [91]	Human	Liver	Organic amines	8, 15, 23, 32	69 h
2005	Dang Yonghui et al. [92]	Rat	Skeletal muscle	pH value	24–28	24 h
2008	Jin Junfeng et al. [93]	Human	Bone marrow of sternum	Lipid	32	9 d
2008	Yang Tiantong et al. [94]	Rabbit	Brain	Nacetylaspartate(Naa)/creatine(Cr), choline(Ch)/Cr	10, 30	24 h
2008	Yang Tiantong et al. [95]	Rabbit	Brain	Naa, Ch, Cr	30	24 h
2012	Yang Tiantong et al. [96]	Rabbit	Blood of right ventricle	pH value	10, 15, 20, 25, 30, 35	66 h

Throughout the world, forensic scientists have employed thanatochemical methods for PMI determination. To this end, they have studied a range of substances, including urea nitrogen, creatinine, uric acid, potassium, magnesium, sodium, chloride, calcium, hypoxanthine levels in postmortem serum, vitreous humour, cerebrospinal fluid and even pericardial fluid [4, 86–90]. Chinese researchers have also explored thanatochemistry as a means to estimate PMI.

Zhang et al. found that the organic amines produced during putrefaction under temperatures of 8 °C, 15 °C, 23 °C and 32 °C increased as PMI extended until attaining their peak values [91]. Meanwhile, other amino acid components decreased gradually over time after death. Dang et al. reported that the pH of rat quadriceps femoris muscle decreased with increasing PMI (within 12 h), which provided a useful linear negative correlation [92]. The formula describing this relationship between PMI and pH value is $y=115.499-11.7x(R^{2}=0.662,P<0.01)$. Jin et al. found that the amount of lipid decreased gradually in a linear relationship with time after death, reaching a minimum value at 10 days post mortem [93]. In a different study, Yang et al. reported that the Naa/Cr ratio decreased, while the Ch/Cr ration increased as the PMI extended through the first 24 h [94]. Using Naa/Cr as the independent variable, the quadratic polynomial regression equation was expressed as

$$y=-0.0020x^{2}-0.0815x+1.4532(R^{2}=0.971)$$

With Ch/Cr as the independent variable instead, the quadratic polynomial regression equation was expressed as

$$y=-0.0024x^{2}+0.0870x+1.1876(R^{2}=0.962)$$

More recently, Yang et al. used a different sample type and found that pH values in the right ventricle correlated with increased PMI at different ambient temperatures ($R^{2}$= 0.974–0.982) [96].

Using thanatochemistry principles, Chinese forensic scholars have constructed regression equations and provided some basic data for the estimation of PMI. Thanatochemical applications for PMI estimation can be problematic, as the compounds of interest are also influenced by environment factors. Scientists have found that the best fluids to study are the vitreous humour and the cerebrospinal fluid, to minimize the effects of the environment. However, these fluids are difficult to obtain, limiting the progress of this approach in the field of forensic practice.

## Other methods

Chinese forensic scholars have also applied a range of novel methods to the estimation of PMI. Wang et al. studied the correlation between PMI and both cholinesterase (ChE) and glutamic oxaloacetic transaminase (GOT) activity in rabbit vitreous humour using spectrophotometry [97]. Results showed initial ChE and GOT activities were approximately 850 and 220 IU/L, respectively, decreasing to 0 IU/L after 54 h post mortem. The relationship can be expressed by simple and multiple regression equations (Table 8).

**Table 8.** Simple and multiple regression equations [97].

Temperature (°C)	Simple and multiple regression equation	Coefficient of determination, $R^{2}$	Significance, *P*
10–15	$\mathrm{PMI}=46.257-0.045X_{\text{ChE}}$	–0.917	0.01
10–15	$\mathrm{PMI}=51.850-0.204X_{\text{GOT}}$	–0.948	0.01
25–30	$\mathrm{PMI}=40.890-0.047X_{\text{ChE}}$	–0.896	0.01
25–30	$\mathrm{PMI}=46.000-0.198X_{\text{GOT}}$	–0.944	0.01
10–15		0.970	0.01
25–30	$\mathrm{PMI}=48.364-0.010X_{\text{ChE}}-0.252X_{\text{GOT}}$	0.959	0.01

**Fig 1.**
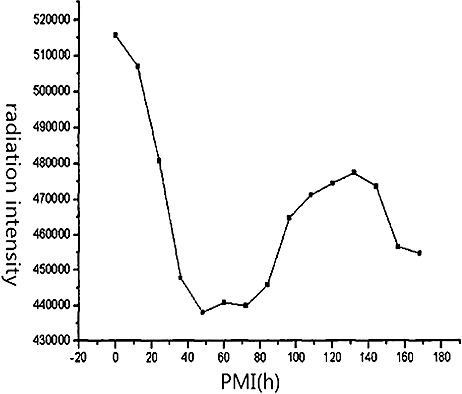
The relationship between radiation intensity and PMI in albino rats [98].

Xue studied the relationship between albino rat imbibitions and PMI, using γ radiation from the autoscaler FH463A [98]. The main result is showed in Figure 1. It demonstrated that the imbibitions of γ radiation may be a useful estimator of PMI.

Mao et al. developed a rapid method for the estimation of PMI using electrical impedance spectroscopy [99]. Linear regression analysis between the maximal absolute value of Im Z″ (capacitive reactance component) and PMI were performed in every group. Results are presented in Table 9. It is demonstrated that the maximum absolute value of Im Z″ (capacitive reactance component) in electrical impedance per sample of each group gradually diminishes as time progresses.

**Table 9.** Linear regression equations [99].

Group	Linear regression equation	Coefficient of determination, $R^{2}$	Significance, *P*
10 °C (spleens in cabinet)	y=−95.517x+865.250	0.956	0.01
10 °C (spleens in vivo)	y=−97.533x+876.819	0.963	0.01
20 °C (spleens in cabinet)	y=−105.713x+926.410	0.980	0.01
20 °C (spleens in vivo)	y=−96.427x+827.497	0.978	0.01
30 °C (spleens in cabinet)	y=−146.786x+1 000.429	0.962	0.01
30 °C (spleens in vivo)	y=−143.085x+976.536	0.964	0.01

Yang et al. investigated the correlation between the changes of oxidation reduction potential (ORP) values of heart blood in rabbits and the PMI at different temperatures after death [100]. Results showed the ORP values were highly correlated with the PMI at each of the temperatures studied (Table 10). As expected, the ORP values increased when the temperature was high, and decreased with lower temperatures.

**Table 10.** Curvilinear regression equations [100].

Temperature (°C)	Curvilinear regression equation	$R^{2}$
10	$y=4.213x^{3}-0.002x^{2}+0.050x+11.026$	0.986
15	$y=6.558x^{3}-0.004x^{2}+0.159x+10.580$	0.983
20	$y=9.005x^{3}-0.006x^{2}+0.210x+10.414$	0.981
25	$y=9.133x^{3}-0.004x^{2}+0.167x+10.794$	0.984
30	$y=0.001x^{3}-0.005x^{2}+0.207x+11.350$	0.982
35	$y=0.001x^{3}-0.002x^{2}+0.380x+11.004$	0.974

Zhang et al. studied the time- and temperature-dependent survival of ovarian oocytes collected from a mouse carcass [101]. Results showed that at a constant temperature, the number of collected germinal vesicle oocytes in the ovary decreased with increasing PMI. Meanwhile, during this time the rate of germinal vesicle breakdown (GVBD) and the first polar body emission (PBE) gradually reduced as the temperature increased.

In more recent years, researchers in other countries have also taken advantage of novel ways for estimating time since death, including GC-MS/MS [73,102,103], micro-computed tomography, mid-infrared microscopic imaging, energy dispersive X-ray mapping [104], LC–MSMS [105], UPLC/Q-TOF MS and SELDI-TOF-MS [106,107]. While these methods are very convenient and efficient for use in the estimation of PMI, but the results obtained are still not precise enough for forensic practice.

## Conclusion

As we have discussed here, there have been numerous attempts by Chinese forensic scholars during the last decades at finding methods to help estimate PMI more accurately. So far, none of them allows us to define the PMI with absolute precision if used alone. Currently, for the early PMI (up to 24 h), the Henssge nomogram is usually applied, complemented by an assessment of hypostasis and rigor mortis, sometimes including consideration of some supra-vital reactions [2]. However, recent Chinese forensic science studies, such as examinations of DNA or RNA degradation and measurements of pH value, have shown potential for new ways to estimate early PMI. There is also more work being done in Chinese research institutes to develop methods for longer PMI estimations, e.g. using entomological methods and spectroscopy techniques. The former is widely considered to be a standard method to estimate longer PMI, but it does have some disadvantages. First, it has no standard for verification and the methodologies for estimation are somewhat subjective, which means outside influencing factors can have a big effect on the resulting PMI estimation. Second, variable seasonal and regional factors also affect the accuracy of long PMI estimation. Conversely, spectroscopy is objectively measured, is convenient, efficient and usually can be performed easily. Therefore, it shows great potential for applications to PMI estimation. However, the obvious drawback is that the sample being measured is always affected by the environment temperature, which is difficult to account for using spectroscopic techniques.

With the continued development of new technology an increasing number of methods have been assessed for the estimation of PMI. Unfortunately, there is still no simple method that can provide a precise estimation of PMI in forensic practice. It appears that using a combination of different methods is the future trend for the estimation of PMI. Regardless of the approach, influential environment factors should always be taken into account when analyzing forensic casework samples.

## Compliance with Ethical Standards

All procedures performed in studies involving human participants were in accordance with the ethical standards of the Human Research Committee of Liaoning Medical University with the 1964 Helsinki declaration and its later amendments or comparable ethical standards.
